# A Call for Inclusion: Children With Intellectual Disabilities in Trauma Treatment Research

**DOI:** 10.1111/jir.70011

**Published:** 2025-08-07

**Authors:** Zackary F. Moore, Anna W. Wright, Carine E. Leslie, Allison D'Aguilar, Ananda B. Amstadter, Ruth C. Brown

**Affiliations:** ^1^ Massey Comprehensive Cancer Center Virginia Commonwealth University Health System Richmond USA; ^2^ Department of Psychiatry Virginia Commonwealth University Richmond USA; ^3^ Department of Psychology Virginia Commonwealth University Richmond USA

**Keywords:** children, inclusion, intellectual disability, posttraumatic stress disorder, randomised controlled trials

## Abstract

**Background:**

Children with intellectual disabilities (ID) experience disparities in mental health care despite experiencing increased exposure to trauma such as physical, emotional and sexual abuse. Studies have suggested that the exclusion of people with ID from medical research may contribute to disparities in health care. It is currently unknown to what extent children with ID are excluded from trauma treatment research.

**Methods:**

All randomised controlled trials (RCTs) (*n* = 62) included in the most up‐to‐date International Society for Traumatic Stress Studies (ISTSS) clinical care guidelines meta‐analysis of posttraumatic stress disorder (PTSD) treatments were reanalysed and coded to determine whether or not children with ID or other developmental disabilities that co‐occur with ID were excluded based on inclusion/exclusion criteria. Articles were double‐coded by the research team. The corresponding authors of the studies were asked to complete a survey rating the likelihood that children with ID would have been eligible for the study.

**Results:**

Of the studies, 61.3% reviewed reported exclusion criteria based on a diagnosis of intellectual disability, cognitive impairment, autism spectrum disorder, organic/neurological conditions, or other related terms (e.g., ‘significant learning difficulties’). Operationalisation of exclusion criteria was sparse. Few studies reported the number of children excluded from trials based on an ID diagnosis.

**Conclusions:**

The evidence base for the ISTSS clinical care guidelines of child PTSD treatment is not representative of children with ID. Improved documentation of inclusion/exclusion criteria, reporting of disability as a demographic characteristic, and inclusion of children with ID in PTSD treatment trials are needed to improve representation of children with ID in PTSD research.

Intellectual disability (ID; or intellectual developmental disorder according to the *Diagnostic and Statistical Manual of Mental Disorders, Fifth Edition, Text Revision* (DSM‐5‐TR) is characterised by significant limitations in core cognitive skills, including the ability to reason, solve problems, plan, think abstractly, make sound judgements and learn both academically and from experience. These cognitive challenges lead to difficulties in adaptive functioning, meaning the individual struggles to meet age‐appropriate expectations for independence and social responsibility across areas such as communication, social engagement, school or work performance and self‐care within home or community environments (American Psychiatric Association 2022). Modern diagnostic approaches have moved beyond narrow IQ‐based definitions, instead emphasising more comprehensive assessments that capture multiple aspects of functioning (Bertelli et al. 2014; Tassé et al. 2016). These assessments focus on deficits in intellectual functions—such as reasoning, problem‐solving, planning and learning—as well as conceptual, social and practical domains of adaptive behaviour. Within the broader umbrella of intellectual and developmental disabilities (IDD), children with ID frequently present with additional co‐occurring neurodevelopmental conditions (e.g., communication disorders, autism spectrum disorder, ADHD) that may further influence their support needs and response to treatment. An estimated 5.76–6.99% of children in the United States have a diagnosis of IDD (Zablotsky et al. 2019).

Research consistently shows that children with ID with and without co‐occurring developmental disabilities, inclusive of both children and adolescents (i.e., <18 years old), experience trauma at significantly higher rates than their same age peers (Berg et al. 2019; Fang et al. 2022; Hoch and Youssef 2020; Jones et al. 2012; McDonnell et al. 2019). For example, Berg et al. (2019) found that children with IDD reported more negative family experiences, regardless of household income or residential stability. McDonnell et al. (2019) also found that children with autism spectrum disorder (ASD) and/or ID were two to three times more likely to experience sexual, physical and emotional abuse, or neglect compared to a population‐based comparison group, even after adjusting for demographic factors. Similarly, a systematic review by Jones et al. (2012) revealed that children with ID (defined as ‘mental or intellectual disability’) are 4.3 times more likely to experience maltreatment than their non‐disabled peers. Fang et al.'s (2022) global meta‐analysis provided further support that children with mental or cognitive impairments face a greater likelihood of abuse or neglect compared to those with *other primary disability types*, such as physical, sensory, or chronic health impairments. While there can be considerable overlap between cognitive and sensory or physical differences (e.g., co‐occurring autism and sensory processing difficulties), Fang et al. (2022) specifically identified cognitive impairment as a high‐risk category when contrasted with physical or sensory disabilities whose core features are not primarily cognitive. Other studies have found children with ID to face an elevated risk for exposure to community violence (Brendli et al. 2022), bullying (Blake et al. 2012) and family violence (Jones et al. 2012; Paquette et al. 2018). Moreover, recent findings indicate that many of these children report experiencing multiple and/or repeated traumatic exposures (i.e., polyvictimisation; Lapshina and Stewart 2021; Vanderminden et al. 2023).

Several studies suggest that children with ID may be more susceptible to the disruptive effects of traumatic events (e.g., posttraumatic stress disorder [PTSD]) than children without ID due, in part, to the compounded effect of differences in cognitive and/or adaptive capacities (e.g., executive function, self‐regulation) and a restricted range of coping skills (Daveney et al. 2019; Mevissen et al. 2016; Rouleaux et al. 2024). Notwithstanding these differences (Hronis et al. 2017), children with ID experience many of the same symptoms of PTSD as their typically developing peers (McCarthy et al. 2016; Mevissen et al. 2014, 2016; Rittmannsberger et al. 2020).

Despite the increased rates and impact of trauma exposure for children with ID, the evidence base of clinical interventions for these children is still scarce (Byrne 2022). Despite the documented impact of trauma on children with ID, a longstanding view has held that they lack the cognitive capacity to engage in or benefit from trauma‐focused treatment (Hronis et al. 2017). This misconception is often compounded by *diagnostic overshadowing*, wherein behavioural challenges are attributed solely to a child's ID rather than recognised as potentially stemming from co‐occurring trauma or mental health concerns (Mason and Scior 2004; Reiss et al. 1982). As a result, children with ID have historically been underrepresented or excluded from trials testing evidence‐based trauma interventions (DeCormier Plosky et al. 2022). Existing clinical trial research on trauma treatment specifically for children with ID is often limited by small, underpowered sample sizes without comparison groups or randomised controlled trials (RCTs; e.g., Byrne 2022). Small studies can be very well designed and demonstrate causality and are invaluable in identifying and addressing complex cases (Hekler et al. 2019), such as people with ID with higher support needs. However, they are not sufficient on their own. Well‐powered and rigorous RCTs are still needed to develop ‘consilient knowledge’ (Hekler et al. 2019), such as those that inform clinical care guidelines and will be needed to overturn the misconceptions that are prevalent in clinical practice. Furthermore, it is widely unknown to what extent children with ID are represented in RCTs for PTSD treatment more generally.

Systematic work has been undertaken to determine the strength of the evidence of the existing PTSD treatments for children (strict criteria for study design and power) and to apply overall recommendations based on the meta‐analyses to guide clinicians on employing the best treatments that work. The International Society for Traumatic Stress Studies (ISTSS) conducted a meta‐analysis that included a review of randomised control trials for child and adult prevention and treatment of PTSD, including psychosocial and pharmaceutical interventions (Forbes et al. 2020). The scoping questions for the meta‐analysis were focused on determining if the given intervention, when compared to treatment as usual, waiting list, no treatment, or other psychological treatments, resulted in a clinically significant reduction of symptoms, improved functioning/quality of life, presence of disorder, or reduced adverse effects. Although most interventions resulted in insufficient evidence to recommend, several interventions were recommended, including child‐focused cognitive‐behavioural therapy for trauma (CBT‐T; strong), group child CBT‐T (emerging), caregiver and child CBT‐T (strong), eye movement desensitisation and reprocessing (EMDR; strong), group psychoeducation (emerging) and parent–child relationship enhancement (emerging) (Forbes et al. 2020).

It is possible that the resulting clinical guidelines also apply to children with ID; however, given that the exclusion of children with ID has been documented in mainstream medical and developmental psychology research (Feldman et al. 2013, 2014), it has yet to be documented the extent to which children with ID are excluded from PTSD RCTs. It is unclear the degree to which the guidelines may generalise to children with ID and PTSD. Identifying populations under‐represented in research is critical for prioritising future research to improve health disparities (American Psychological Association 2003; Safer‐Lichtenstein et al. 2019; Watkins 2012).

The present study aims to fill this gap by examining the extent to which children with ID are specifically included or excluded from participating in PTSD randomised controlled treatment studies and, if so, what justifications or barriers to participation were noted. We hypothesise, based on similar reviews of the medical and developmental literature, that the exclusion of children with ID will be common and that perceptions of the ability to assent, complete written assessments, and the ability to participate in treatment will be cited as barriers to inclusion (Feldman et al. 2013, 2014; Spong and Bianchi 2018). This data is critical for identifying strategies and research to address the obstacles to participation in research and treatment to ensure distributive justice in research and access to mental health care.

## 1 Methods

We performed a systematic re‐analysis of the 62 randomised controlled trials for PTSD treatment studies in children from the ISTSS 2019 meta‐analysis (Forbes et al. 2020; see Supplemental Materials for complete list). No new studies were included, as this review specifically evaluates the representation of children with ID within the evidence base used to inform the current ISTSS clinical care guidelines, which remain central to evidence‐based treatment practices despite being limited to studies published through 2019. In all, 62 articles on trauma treatment for children were reviewed. Forty‐five (72.6%) were psychological treatment studies, 13 (21%) were early psychological intervention studies, two (3.2%) involved pharmacological treatment/intervention studies, and two (3.2%) were non‐psychological and non‐pharmacological treatment studies. The articles were coded in REDCap (Research Electronic Data Capture), a secure Web‐based application designed for building and managing online surveys and research databases (Harris et al. 2009), by two separate research assistants (ZM and AD), and disagreements in coding were solved by the senior investigator (RB) or an advanced psychology graduate student (CL). Study descriptive data extracted from each article included treatment/intervention type, researcher location/country, how many children were screened for the study, how many children were eligible for the study, and how many children began treatment.

### 1.1 Determination of Inclusion and Exclusion

The methods section of each study was reviewed for inclusion/exclusion criteria. Exclusion based on ID was indicated by the use of specific diagnostic labels, including intellectual disability/disorder (ID), mental retardation, or learning disability (if the study was conducted in the United Kingdom). We also indicated whether other ‘related conditions’ (i.e., developmental disabilities or conditions) that may systematically exclude or over‐represent ID were used as exclusionary criteria: Specific Learning Disability (SLD; US studies), Cognitive Impairment (CI), Organic Brain Dysfunction/Disorder, Developmental Disorder/Disability/Delay (DD), Autism/Asperger's/Pervasive Developmental Disorder (ASD), Genetic conditions (e.g., Down syndrome, Fragile X syndrome), and Other (used to capture variations in terminology that may or may not be specific to DSM or ICD codes). This included terminology such as ‘severe learning problems’, which may or may not be limited to those with a diagnosis of ID, SLD, or other DDs. Articles were also coded based on whether or not the study set specific IQ or cognitive testing criteria, whether or not the study reported the number of children excluded due to ID or related conditions if they were excluded, and other reasons for exclusion (e.g., behavioural disturbance/risk, presumed poor adherence to procedures or ability to benefit, illiteracy, communication/language deficits, requiring written consent), and whether or not recruitment procedures likely systematically excluded children with ID. Because it has historically been common practice to exclude patients with any comorbid conditions in treatment outcome studies (e.g., co‐occurring anxiety or depression), we also coded whether or not the study excluded any other comorbid conditions, excluded some comorbid conditions but included others, did not exclude any other conditions, and did not report exclusion based on comorbidity. Although a study that excluded any co‐occurring psychiatric condition may have excluded someone with an ID diagnosis, these were not coded as excluding ID specifically unless they mentioned one of the codes mentioned above. Articles were also coded based on whether or not the study reported *including* children with ID.

### 1.2 Author Survey

Due to the aforementioned limitations to reporting of inclusion/exclusion criteria related to ID, the survey was used to gather additional information that could not be extracted from the manuscript. We emailed the corresponding authors to supplement the information extracted from the published articles, sending them a link to a short survey through REDCap, an online survey and data capture platform (Harris et al. 2009). Authors were sent up to three email requests. Authors were asked, ‘To the best of your knowledge, were people with the following types of intellectual or cognitive impairments ELIGIBLE to participate in the study’. They rated the following conditions separately: mild ID, moderate ID, severe/profound ID, other DD, including autism spectrum disorders (ASD), and other forms of cognitive impairment, such as traumatic brain injury (TBI). Respondents used a five‐point scale ranging from definitely eligible, probably eligible, unsure, probably ineligible and ineligible. An optional field for comments was also included.

### 1.3 Data Analysis Plan

Analyses were conducted using the R Statistical language (version 4.4.2; R Core Team, 2024) on Windows 10 x64 (build 19 045). A descriptive table and figure were prepared using packages ‘gtsummary’ (version 1.7.2; Sjoberg et al. 2021) and ‘likert’ (version 1.3.5; Bryer and Speerschneider 2016). Inter‐rater reliability was assessed using Gwet's AC1 (Gwet 2014) with the ‘irrCAC’ package (version 1.0; Gwet 2019).

## 2 Results

### 2.1 Reported Inclusion and Exclusion

Overall, agreement ranged from AC1 = 0.66 (95% CI [0.50, 0.83], *p* < 0.001) to AC1 = 0.98 (95% CI [0.91, 1.00], *p* < 0.001). According to conventional benchmarks (Landis and Koch 1977), these coefficients suggest reliability ranging from substantial (AC1 ≈ 0.66–0.78) to near‐perfect (AC1 ≈ 0.92–0.98). All *p* values were highly significant (*p* < 0.001), indicating that observed agreement exceeded what would be expected by chance for every variable.

Based on reported exclusion criteria, children with ID or related conditions were excluded from 61.3% (*n* = 38) of studies (see Table 1). The most common of these were intellectual disability/mental retardation (27% of all studies) or ASD (19% of all studies). Twenty‐one percent (*n* = 13) of the studies had other exclusionary criteria that likely resulted in screening out children with ID, including the following: traumatic brain injury, neurological disorder, head injury/trauma, ‘not mentally competent’, or learning problems/difficulty. These findings indicate that the majority of RCTs in the sample explicitly excluded children with ID or ASD (with or without specifying co‐occurring ID) revealing a significant representation gap for this population in mainstream child PTSD research.

**TABLE 1:** Frequency of reported exclusion criteria related to intellectual or developmental disabilities.

Exclusion criteria	*n*	%
Intellectual disability	17	27.0
Specific learning disability	0	0.0
Organic brain disorder	3	4.8
Cognitive impairment	4	6.5
Developmental disability	4	6.5
Autism spectrum disorder	12	19.0
Genetic disorders	0	0.0
Other	13	21.0
One or more of the above categories	38	61.0

*Note: N* = 62. Intellectual disability included mental retardation or ‘learning disability’ if the study was conducted in the United Kingdom. Developmental disability included developmental delay. ‘Other’ included variations on terms that could not be specifically classified, such as ‘significant learning difficulties’, but implied a diagnosed or perceived intellectual or developmental disability.

Of the articles reviewed, 55 (90.2%) did not report a specific IQ or cognitive testing criteria. Three (4.9%) articles excluded anyone with ID based on IQ, two (3.3%) articles allowed mild ID or equivalent (meaning IQ ranging from 50 to 55 to approximately 70), and one (1.6%) article allowed moderate ID or equivalent (meaning IQ ranging from 35–40 to 50–55). One study set the criteria at an IQ estimated to be ≤80, which would have also excluded those with borderline intellectual functioning. These findings show that nearly all studies did not use explicit IQ‐based criteria, and those that did typically set restrictive thresholds, suggesting an inconsistent and often narrow approach to determining ID‐related eligibility.

In a review of the 38 studies with ID‐related exclusion criteria, only 9 (23.7%) provided a CONSORT diagram or specifically reported the numbers and reasons for excluded children with ID, with the number of excluded children with ID ranging from 0 to 9 per study. This constituted 0 to 5.17% of the screened participants in each study, amounting to 30 children out of 2481. Additionally, two studies mentioned exclusions for unspecified reasons, which may or may not have included children with ID. These results suggest that most studies neither documented the specific exclusion of children with ID nor tracked their numbers systematically, making it difficult to determine the true extent of ID/IDD exclusion within these trials.

In our analysis of exclusion criteria for children with ID, we categorised the reasons provided by the 23 articles that offered justification for exclusion. The reasons, ordered by frequency, are as follows: both behavioural disturbance and risk, and communication/language deficits affecting participation, each cited in 30.4% of these articles; illiteracy or inability to complete assessments, mentioned in 26.1%; required written assent, noted in 8.7%; and equal mentions (4.3% each) for physical disabilities, presumed poor adherence to procedures or ability to benefit, and exclusion by mental status exam. These findings show that behavioural disturbance, communication deficits, and illiteracy or inability to complete assessments were the most frequently cited reasons for excluding children with ID, reflecting common concerns about their potential to participate meaningfully in these studies.

### 2.2 Author Survey

A total of 42 corresponding authors (67.7% of the articles reviewed) responded to the survey, with 40 completing the survey questions (two responded, indicating they declined to answer). Thirty (75.0%) identified themselves as the principal investigator of the study, six (15.0%) were the study supervisor, and nine (22.5%) were the study coordinator (some selected multiple roles). Additional responsibilities they endorsed being involved with included recruitment/enrollment (12.5%), screening (10.0%), data collection (12.5%), treatment (20.0%) and other responsibilities (12.5%). Of those who responded, 27 were authors of papers that reported exclusion of ID or related conditions, and 15 were authors of papers that did not report exclusion. The likelihood of responding to the survey was not significantly associated with whether the exclusion of ID was reported in the manuscript (B = 0.39, 95% CI [−0.71, 1.48], *p* = 0.484).

**FIGURE 1:**
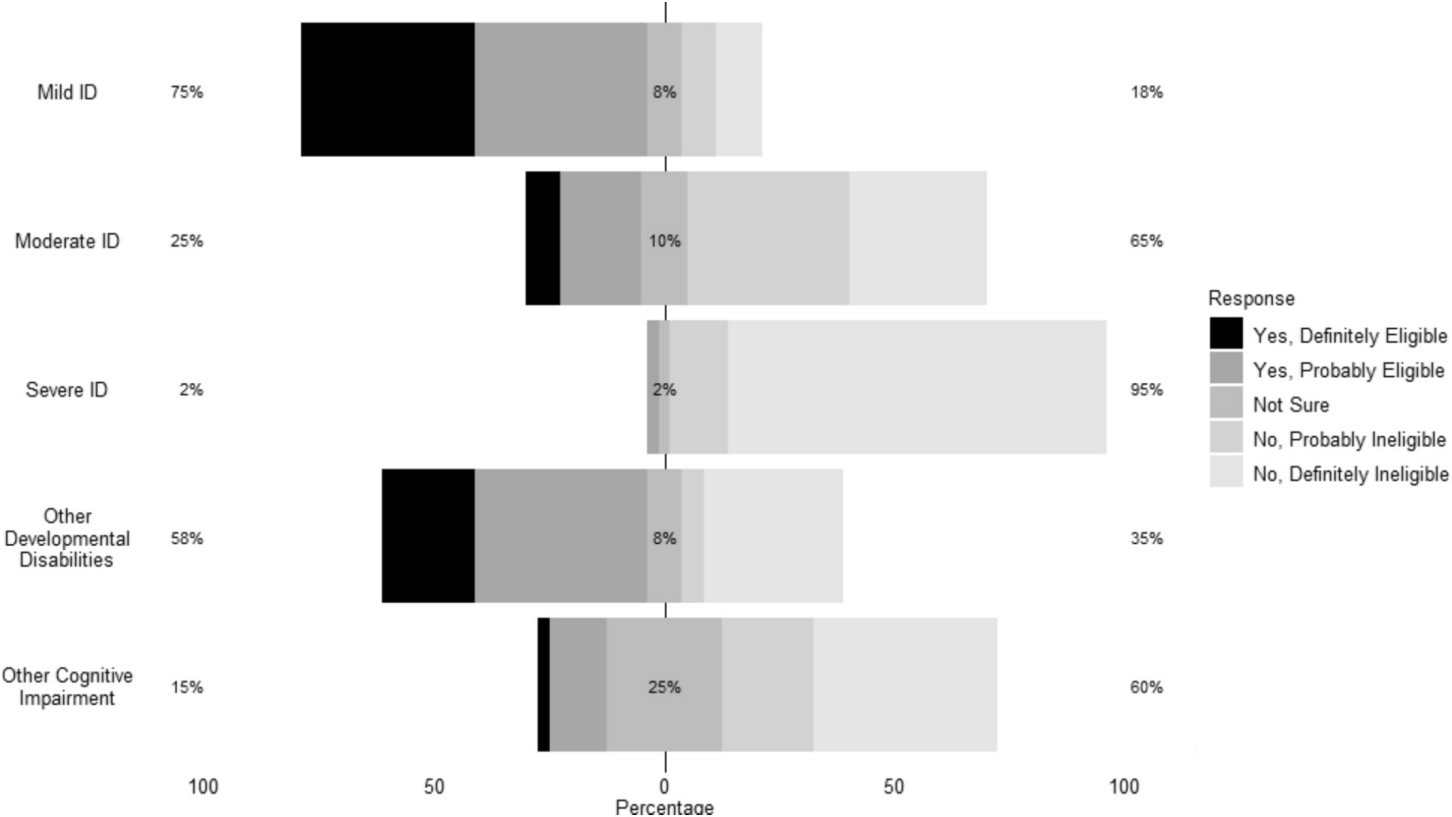
Author‐reported eligibility for study inclusion based on intellectual and/or developmental disability. *Note*. *n* = 42. Authors of 42 studies rated whether, to the best of their knowledge, children with the following intellectual and/or developmental disabilities would have been eligible in the referenced study. Percentages on the left *Y*‐axis reflect the combination of ‘Yes, Definitely Eligible’ and ‘Yes, Probably Eligible’ responses. Percentages on the right *Y*‐axis reflect the combination of ‘No, Definitely Ineligible’ and ‘No, Probably Ineligible’ responses.

Corresponding authors were asked whether or not, to the best of their knowledge, children with mild ID, moderate ID, severe or profound ID, other developmental disabilities, or other cognitive impairments would have been eligible for participation. Response rates for the likelihood of eligibility for each descriptive group are reported in Figure 1. Most respondents (75%) indicated that children with *mild* ID were ‘definitely’ or ‘probably’ eligible. In contrast, only 25% reported the same for *moderate* ID, with a majority (65%) rating them as ‘probably’ or ‘definitely’ ineligible. For *severe* ID, 95% rated them as ‘probably’ or ‘definitely’ ineligible, and no respondents indicated ‘definitely eligible’. Reports for children with *other developmental disabilities* (e.g., autism spectrum disorders) fell between the ranges observed for mild and moderate ID, with 58% indicating ‘definitely’ or ‘probably’ eligible and 35% indicating ‘probably’ or ‘definitely’ ineligible. Finally, for children with other cognitive impairments (e.g., traumatic brain injury), there was a wider degree of uncertainty; approximately 15% rated them as ‘definitely’ or ‘probably’ eligible, 25% were ‘not sure’, and 60% rated them as ‘probably’ or ‘definitely’ ineligible. These findings suggest that while mild ID might be perceived as less of a barrier, children with moderate or severe ID or other complex cognitive impairments were generally seen as unlikely to be eligible.

Authors were given the opportunity to provide optional free‐text comments regarding eligibility and inclusivity of the study. Twenty‐four authors provided additional comments, and responses ranged from justification for exclusion to elaboration on the screening process and how this would influence inclusivity. Most narrative responses indicated that inclusion or exclusion was based on a child's ability to engage in the treatment and/or complete study measures but did not exclude based on a diagnosis of ID. Several noted that they did not conduct IQ testing, so they could not verify the rate at which children diagnosed with ID were included or excluded. A few said that participants for their study were recruited from the public school system. This recruitment characteristic led to conflicting conclusions across responding authors; however, some noted that due to public school recruitment, they believed children with ID could have been included, while one said that children with moderate to severe intellectual disability were likely excluded from the schools where recruitment occurred. Multiple authors' responses indicated that they were unsure of the definition of ID or conflated ID with ASD.

## 3 Discussion

The purpose of this study was to examine the inclusion and exclusion criteria of child PTSD RCTs to determine the extent to which children with ID or related neurodevelopmental conditions were represented in the evidence base that formed the ISTSS clinical care guidelines. An essential aspect of identifying ‘what works for whom’ is the identification of the gaps in ‘for whom’ we have effective treatments. Children with ID are vulnerable to higher rates of trauma and PTSD compared to children without ID as well as children with other IDDs (e.g., ASD) without ID (Brendli et al. 2022; Fang et al. 2022; Jones et al. 2012) highlighting the need for representation in PTSD treatment research. All 62 articles reviewed in the most recent 2019 ISTSS meta‐analysis of trauma treatment studies were reviewed and coded based on their inclusion and exclusion criteria, study methods and corresponding author reports of participant eligibility.

Our findings suggest that a majority of PTSD RCT studies (61%) explicitly excluded children with IDor a related developmental disabilities. Unfortunately, for the remaining studies that did not list explicit exclusion criteria, it cannot be assumed that children with ID were included or excluded. Additionally, most of the studies did not provide a reason for the exclusion of children with ID. For example, studies often simply listed ‘severe cognitive impairment’ in a list of exclusion criteria rather than saying, ‘Children with severe cognitive impairment were excluded due to concerns about the validity of outcome measures’. Notably, nearly one‐third of the studies citing a reason for exclusion mentioned behavioural disturbance or perceived risk.

There are several potential explanations for the frequent exclusion of children with ID without further justification that warrants careful consideration. One possibility is that pervasive training gaps in ID among mental health providers result in a lack of knowledge or clinical competence, or inaccurate assumptions about the ability of any child with ID to participate in research or psychological treatment (Brooker et al. 2014; Pelleboer‐Gunnink et al. 2017; Werner et al. 2013; Werner and Stawski 2012). It may reflect challenges in adapting the standardised treatment protocols to address these atypical symptom presentations or to accommodate children with more severe ID, who may be more likely to engage in aggressive or self‐injurious behaviours (Emerson et al. 2001; Poppes et al. 2016). Another possibility is diagnostic overshadowing, whereby clinicians or researchers attribute challenging behaviours to the child's ID rather than considering that these behaviours might be a symptom of PTSD (Rittmannsberger et al. 2020) which may be modifiable through PTSD treatment. Nevertheless, rather than justifying exclusion, these complexities should galvanise efforts to adapt and develop trauma interventions that are accessible and effective for children across the full spectrum of ID, including those with severe impairments and co‐occurring challenging behaviours, as we elaborate later in our recommendations for future research.

It was rare for studies that explicitly excluded children with ID to report the number of children with ID who were excluded from participating or the specific diagnoses they may have had (e.g., ASD with or without ID). Most studies did not set specific IQ or cognitive testing criteria for participants. Among the studies that did not explicitly exclude children with ID or related conditions, we cannot be sure of the rates of inclusion of children with ID in these studies, as their participation is not expressly stated. Indeed, no studies reported explicitly including children with ID in the trials. The author survey found that, in retrospect, most corresponding authors believed children with *mild* ID would have been ‘definitely’ or ‘probably’ eligible for their trials—although this may reflect current perspectives rather than those held at the time the original studies were conducted. The likelihood of authors reporting eligibility decreased with increasing severity of ID, highlighting the need to further explore strategies for including children with higher levels of support needs in PTSD trials.

Findings are consistent with studies utilising similar methodology to explore the inclusion and exclusion of people with ID in the medical (Feldman et al. 2014; McDonald et al. 2022; Spong and Bianchi 2018) and child development (Feldman et al. 2013) literature. For example, Feldman et al. (2013) surveyed child development research for the inclusion of children with disabilities, including ID, in studies published over a span of 15 years. The authors reported that 70% of articles' did not specify whether these children were included, whereas of the articles that did, 66.7% explicitly excluded children with ID. The majority (74%) of studies did not provide any justification for the exclusion (Feldman et al. 2013). This suggests that the PTSD treatment field is not unique yet reflects a common practice of exclusion in the medical and social sciences.

### 3.1 Opportunities to Increase Inclusion of Children With ID in PTSD Research

One goal of this review is to highlight opportunities to close important evidence gaps. Altogether, the results of this study suggest that the evidence base for children's PTSD treatment is not representative with regard to ID status. Improved documentation of inclusion/exclusion criteria, reporting of disability as a demographic characteristic and inclusion of children with ID in PTSD treatment trials are needed to improve mental health disparities experienced by children with ID. We discuss these findings in turn, along with recommendations and examples for how the inclusion of children with ID can be maximised in the future.

#### 3.1.1 Precise and Reproducible Referral and Inclusion/Exclusion Criteria

Although some studies explicitly stated the presence of an ID diagnosis as an exclusion criterion, others used vague descriptions such as ‘severe cognitive impairment’, which may be interpreted differently by different people. For instance, is an IQ that is two standard deviations below the norm (e.g., mild ID) considered a severe impairment, or is this referring to ‘severe intellectual disability’ (i.e., four or more standard deviations below)? IDDs are a heterogenous class of disorders. Even within a category, such as people with mild ID, there is great variability in specific skills needed to engage in treatments such as receptive and expressive language, abstract reasoning, reading and writing (Fisch et al. 2012). Additionally, within youth with ASD, there is a wide range of support needs depending on whether they have co‐occurring ID, verbal communication and degree of social relatedness that will have significant ramifications for the extensiveness of adaptations to a standard treatment protocol. This suggests that setting predetermined criteria, such as an IQ score or adaptive behaviour testing, may not effectively distinguish between who is likely to benefit from those who are not, especially in the absence of empirical studies to establish such a criterion. Indeed, there are no studies examining the influence of IQ scores on the ability to participate in research, generally, or psychotherapy, specifically. Nor are there studies on the influence of IQ scores on the likelihood of benefitting from psychological or pharmaceutical interventions. These represent high priority research areas to fill these methodological gaps.

At the same time, leaving the decision to clinical judgement poses challenges. Given that most mental health providers do not receive training in ID or IDD mental health services (Pinals et al. 2022; Werner et al. 2013; Werner and Stawski 2012), there are likely to be misconceptions about their ability to participate in therapy, which may introduce uncontrolled bias in the screening and enrollment process.

One potential for improving pre‐screening for inclusion/exclusion criteria that may be less dependent on IQ or adaptive testing is the identification of necessary pre‐requisite skills for engaging in psychotherapy. For example, Lickel et al. (2012) conducted a study of children with and without ASD, assessing the prerequisite skills of emotion recognition, discrimination between thoughts, feelings and behaviours, and cognitive mediation, and found comparable rates of all but emotion recognition. Other studies of adults with ID have also tested assessments of prerequisite skills and the effectiveness of interventions in improving these skills. However, their associations with treatment outcomes are lacking and continue to be an important area of future research (Cooney et al. 2017). Ideally, these pre‐requisite skills would not be used as a simple inclusion/exclusion criteria, but as a routing criteria for pre‐treatment remedial skill development or planned adaptation (e.g., enhanced parental involvement, simplified language, visual aids, e.g., Hoover et al. 2024; Schipper‐Eindhoven et al. 2024).

#### 3.1.2 Collection and Reporting of ID as Demographic Information

A small minority of studies reported the number of children screened out of studies based on disability. Thus, it is unknown the extent to which children with ID may or may not have presented for participation, been screened out, enrolled, etc. Increased recognition of the importance of collecting and reporting demographic information related to sex, gender, race, ethnicity and socioeconomic status has been critical in identifying limitations to generalisability for other minoritised and underrepresented populations, improving the representation of study samples, and developing tailored interventions. Standards around documentation of disability status are needed to improve awareness and inclusion of children with ID in research generally (Feldman et al. 2013; Loeb 2013; Spong and Bianchi 2018) and trauma research.

#### 3.1.3 Psychotherapy Research for Children With ID Is Possible

Despite the widespread exclusion from research, studies exist that demonstrate that children with ID can participate in psychotherapy, complete symptom rating scales and assent to participate in research with appropriate accommodations.

##### 3.1.3.1 Inclusive Assessment

Inclusion criteria for the ISTSS meta‐analysis required that studies used standardised measures of PTSD symptoms using either the clinician or self‐report assessments and that at least 70% of study participants meet partial or full DSM or ICD criteria for PTSD using either clinician assessment or self‐report above a clinical threshold of a validated measure. There are concerns about the validity of self‐report assessments with children with ID, especially children with higher support needs, and few clinicians receive training in conducting clinical interviews with children with ID. Furthermore, there are concerns that DSM or ICD criteria may not adequately capture the symptom presentation of children with ID, particularly those with greater cognitive or communication limitations (Fletcher et al. 2016; Mazza et al. 2020). As a result, children with ID may be more likely to be excluded from mainstream RCTs on the bases of measurement or diagnostic concerns. However, there is emerging evidence that children with ID can complete assessments that are adapted. For example, studies by Mevissen et al. (2014, 2016) examining adapted clinical assessments (i.e., the Anxiety Disorders Interview Schedule for Children—PTSD of children with mild to borderline ID who had experienced traumatic events found that, with accommodations, all children could complete the assessments and many qualified for a diagnosis of PTSD following standard diagnostic criteria. However, it should be noted that children with ID with more intensive support needs may experience atypical symptoms of PTSD, which can resemble symptoms of psychosis, so careful assessment is needed in these cases (Mevissen et al. 2016). Future research is needed to further validate adapted self‐report or clinical diagnostic interviews for children across the spectrum of ID, and mainstream research should consider allowing substitution of these adapted measures or diagnostic criteria (e.g., DC‐LD or DM‐ID; Fletcher et al. 2016; Mazza et al. 2020) to facilitate inclusion in these studies.

##### 3.1.3.2 Inclusive Treatment

Although limited, studies have found that trauma‐focused CBT, exposure therapy and EMDR therapy can be used with children and adults with ID and/or ASD (D'Amico et al. 2022; Hoover et al. 2024; Karatzias et al. 2019; Keesler 2020) with minor modifications to standard protocols. The most common modifications made to psychotherapy protocols for people with ID include greater caregiver involvement, simplified language, use of visual aids and increased repetition and practice of content (Byrne 2022; Cooney et al. 2017; Hassiotis et al. 2013; McCabe et al. 2006; Mevissen et al. 2011; Schipper‐Eindhoven et al. 2024). Modified EMDR storytelling has also been successfully used in adults with severe ID (Hoogstad et al. 2024). It has been found that failure to tailor the protocol to the individual needs of children with ID may result in diminished effectiveness (Holstead and Dalton 2013). The detriments of rigid adherence to treatment protocols (Hogue et al. 2008) and the need to individually tailor treatments to individual patients have long been recognised (e.g., (Norcross and Wampold 2011)), and so modifications for children with ID are consistent with existing conceptualisations of flexible implementation (Kendall 2021).

##### 3.1.3.3 Inclusive Research

In alignment with the principles of Universal Design, efforts to modify research and intervention protocols to include children with ID would benefit all patients. These effects have been observed in the built environment (Imrie 2012), product development (Sangelkar et al. 2012), digital health (Henni et al. 2022) and education. Universal Design principles have also been applied to biomedical research and often involve ensuring that materials are accessible (like using Braille or screen‐reader‐friendly formats, plain language and visual supports), adopting inclusive communication methods (such as sign language interpretation or augmented communication devices), ensuring physical accessibility of research spaces (Rios et al. 2016; St. John et al. 2022; Williams and Moore 2011). This approach not only benefits people with disabilities but also enhances the overall inclusivity and relevance of the research. Research finding improved outcomes for all students with the implementation of universal design of learning (for review, see Capp 2017) and inclusion of special education students in mainstream classrooms (for review, see Ruijs and Peetsma 2009) is likely to serve as a model for universal design of mental health and trauma treatment research.

#### 3.1.4 Institutional Policies That Protect Through Research Rather Than From Research

Given the history of abuse of people with ID in research (Feudtner and Brosco 2011), researchers and/or internal review boards may have restricted inclusion out of caution (Carlson 2013; McDonald et al. 2008, 2017). While it is essential to be cautious regarding research on children with ID, it is possible to be overcautious and exclude children with ID from research inadvertently they could have otherwise benefitted from and participated in with little risk (Carlson 2013; Shepherd et al. 2019; St. John et al. 2022). Increasing diversity and access for children with ID to research will require interventions at multiple levels within research institutions. Examples of interventions may include expanding diversity curriculum in graduate and medical education programs to include disability generally and ID specifically to ensure that clinicians and researchers have the necessary skills to include children with ID (Dagnan et al. 2018; Hemm et al. 2015; Ouellette‐Kuntz et al. 2003; Shakespeare et al. 2009; Shakespeare and Kleine 2013), and evaluating policies of internal review boards to support inclusion while providing appropriate protections (Feudtner and Brosco 2011; Shepherd 2016; St. John et al. 2022).

Increased research and funding for research (McDonald et al. 2022) to enhance the participation of children with ID in research will be necessary to close gaps (McDonald et al. 2022). As researchers, institutions and funding agencies strive to increase the diversity and inclusivity of research participants (Landry et al. 2018; Mamun et al. 2019), we must recognise disability as a form of diversity and as an under‐represented and under‐served population (Olkin 2002; Sabatello 2019; Swenor and Deal 2022). Indeed, the recent decision to recognise people with disabilities as a health disparity population by the National Institute of Minority Health and Health Disparities is likely to increase awareness and funding for the inclusion of people with intellectual disabilities in research and treatment studies (National Institutes of Health 2023).

#### 3.1.5 Limitations

The implications of this study should be viewed in light of several limitations. First, the articles we reviewed were limited to the studies included in the 2019 ISTSS meta‐analysis, which was scoped to determine recommendations for evidence‐based treatments based on randomised controlled trials of PTSD treatments (Forbes et al. 2020). It did not include case studies, feasibility studies, single‐arm studies, open trials or treatments for other stress‐related disorders. Future research should replicate this study using a systematic search and review approach so that a more complete sense of how often children with ID are excluded from trauma treatment study research can be achieved. Finally, we did not extend our search to include studies published after the 2019 ISTSS review, so it is unknown whether newer trials have improved ID inclusion; future systematic reviews should incorporate more recent RCTs to determine if inclusion practices are evolving.

Although this study found gaps in inclusion and a lack of clear documentation of inclusion/exclusion criteria related to ID, we cannot infer the causes of exclusion. Future research will need to explore the causes of these gaps. Potential explanations include a lack of awareness, which may stem from the segregation of people with ID in institutions, group homes and contained classrooms, diagnostic overshadowing, misperceptions about the ability of children with even mild ID to participate in psychological treatments or complete outcome measures, misperceptions about the ability of children with ID to assent to research, or awareness of how to make research more accessible to underrepresented communities (e.g., easy read advertisements) (Swenor and Deal 2022). Furthermore, it needs to be clarified the extent to which these barriers stem from investigators, internal review boards (Shepherd et al. 2019) and more considerable systemic barriers (e.g., siloing of service systems and referral networks). For example, many studies rely on patient referrals from other clinicians, social workers, child welfare services, etc. If referral sources are biased based on diagnostic overshadowing or about who is likely to be able to participate or benefit from treatment, children with ID may be never or rarely present to the study team.

## 4 Conclusion

Children with ID are a population that is highly susceptible to trauma and would benefit significantly from increased research on the effectiveness of PTSD treatments and subsequent dissemination of evidence‐based clinical care. Despite this, children with ID are often either excluded from studies focused on PTSD treatment or their inclusion/exclusion is not described. The findings of this study suggest that clinical research on PTSD treatment has become split into two silos: RCTs that exclude children with ID and a separate, smaller literature focused exclusively on specialised interventions for individuals with ID. While it is true that certain subgroups—such as children with co‐occurring autism and intellectual disability or those with severe intellectual disability—often require more targeted adaptations, there is a strong likelihood that many children with mild to moderate intellectual disability could benefit from mainstream therapies if provided with accommodations that address their cognitive or communication needs. Consequently, the field should prioritise expanding eligibility criteria in mainstream PTSD and psychotherapy trials to ensure broader access to evidence‐based interventions in routine practice, while also continuing to develop and rigorously test specialised treatments for those who need more extensive modifications.

## Conflicts of Interest

The authors declare no conflicts of interest.

## Supporting information


**Data S1:** Supporting information.

## Data Availability

The data that support the findings of this study are available from the corresponding author upon reasonable request.
