# Supplementary material for: A Call for Inclusion: Children With Intellectual Disabilities in Trauma Treatment Research
Source: J Intellect Disabil Res. 2025 Aug 7;69(11):1272–83. doi: 10.1111/jir.70011 (PMC12576385; doi:10.1111/jir.70011)
Supplement: Supplementary file 1 — Data S1: Supporting information. [file JIR-69-1272-s001.docx]

**Supplemental Materials**

**References of Studies Included in the ISTSS Meta-Analysis of Child PTSD Treatment Studies**

**Ahmad, A.**, Larsson, B., & Sundelin-Wahlsten, V. (2007). EMDR treatment for children with PTSD: Results of a randomized controlled trial. Nordic Journal of Psychiatry, 61, 349–354.

**Ahrens, J.**, & Rexford, L. (2002). Cognitive processing therapy for incarcerated adolescents with PTSD. Journal of Aggression, Maltreatment & Trauma, 6, 201–216.

**Auslander, W.**, McGinnis, H., Tlapek, S., Smith, P., Foster, A., Edmond, T., & Dunn, J. (2016). Adaptation and implementation of a trauma-focused cognitive behavioral intervention for girls in child welfare. American Journal of Orthopsychiatry, 87, 206–215.

**Berger, R.**, & Gelkopf, M. (2009). School-based intervention for the treatment of tsunami-related distress in children: A quasi-randomized controlled trial. Psychotherapy and Psychosomatics, 78, 364–371.

**Berkowitz, S. J.**, Stover, C. S., & Marans, S. R. (2011). The Child and Family Traumatic Stress Intervention: Secondary prevention for youth at risk of developing PTSD. Journal of Child Psychology and Psychiatry, 52(6), 676–685. <https://doi.org/10.1111/j.1469-7610.2010.02321.x>

**Catani, C.**, Kohiladevy, M., Ruf, M., Schauer, M., Elbert, T., & Neuner, F. (2009). Treating children traumatized by war and Tsunami: A comparison between exposure therapy and meditation-relaxation in North-East Sri Lanka. BMC Psychiatry, 9, Article 22. <https://doi.org/10.1186/1471-244X-9-22>

**Celano, M.**, Hazzard, A., Webb, C., & McCall, C. (1996). Treatment of traumagenic beliefs among sexually abused girls and their mothers: An evaluation study. Journal of Abnormal Child Psychology, 24, 1–17.

**Chemtob, C. M.**, Nakashima, J., & Carlson, J. G. (2002). Brief treatment for elementary school children with disaster-related posttraumatic stress disorder: A field study. Journal of Clinical Psychology, 58(1), 99–112. <https://doi.org/10.1002/jclp.1131>

**Chen, Y.**, Shen, W. W., Gao, K., Lam, C. S., Chang, W. C., & Deng, H. (2014). Effectiveness RCT of a CBT intervention for youths who lost parents in the Sichuan, China, earthquake. Psychiatric Services, 65, 259–262.

**Cohen, J. A.**, & Mannarino, A. P. (1998). Interventions for sexually abused children: Initial treatment outcome findings. Child Maltreatment, 3, 17–26.

**Cohen, J. A.**, Mannarino, A. P., & Iyengar, S. (2011). Community treatment of posttraumatic stress disorder for children exposed to intimate partner violence: A randomized controlled trial. Archives of Pediatrics & Adolescent Medicine, 165, 16–21.

**Cox, C. M.**, Kenardy, J. A., & Hendrikz, J. K. (2010). A randomized controlled trial of a web-based early intervention for children and their parents following unintentional injury. Journal of Pediatric Psychology, 35(6), 581–592.

**Damra, J. K. M.**, Nassar, Y. H., & Ghabri, T. M. F. (2014). Trauma-focused cognitive behavioral therapy: Cultural adaptations for application in Jordanian culture. Counselling Psychology Quarterly, 27, 308–323.

**de Roos, C.**, van der Oord, S., Zijlstra, B., Lucassen, S., Perrin, S., Emmelkamp, P., & de Jongh, A. (2017). Comparison of eye movement desensitization and reprocessing therapy, cognitive behavioral writing therapy, and wait-list in pediatric posttraumatic stress disorder following single-incident trauma: A multicenter randomized clinical trial. Journal of Child Psychology and Psychiatry, 58(11), 1219–1228. <https://doi.org/10.1111/jcpp.12768>

**Deblinger, E.**, Lippman, J., & Steer, R. (1996). Sexually abused children suffering posttraumatic stress symptoms: Initial treatment outcome findings. Child Maltreatment, 1, 310–321.

**Deblinger, E.**, Stauffer, L. B., & Steer, R. A. (2001). Comparative efficacies of supportive and cognitive behavioral group therapies for young children who have been sexually abused and their nonoffending mothers. Child Maltreatment, 6(4), 332–343. https://doi.org/10.1177/1077559501006004006

**Diehle, J.**, Opmeer, B. C., Boer, F., Mannarino, A. P., & Lindauer, R. J. (2015). Trauma-focused cognitive behavioral therapy or eye movement desensitization and reprocessing: What works in children with posttraumatic stress symptoms? A randomized controlled trial. European Child & Adolescent Psychiatry, 24, 227–236.

**Ertl, V.**, Pfeiffer, A., Schauer, E., Elbert, T., & Neuner, F. (2011). Community-implemented trauma therapy for former child soldiers in Northern Uganda: A randomized controlled trial. JAMA, 306(5), 503–512. <https://doi.org/10.1001/jama.2011.1060>

**Foa, E. B.**, McLean, C. P., Capaldi, S., & Rosenfield, R. (2013). Prolonged exposure vs supportive counseling for sexual abuse-related PTSD in adolescent girls: A randomized clinical trial. JAMA, 310, 2650–2657.

**Ford, J. D.**, Steinberg, K. L., Hawke, J., Levine, J., & Zhang, W. (2012). Randomized trial comparison of emotion regulation and relational psychotherapies for PTSD with girls involved in delinquency. Journal of Clinical Child & Adolescent Psychology, 41(1), 27–37. <https://doi.org/10.1080/15374416.2012.632343>

**Gilboa-Schechtman, E.**, Foa, E. B., Shafran, N., Aderka, I. M., Powers, M. B., Rachamim, L., Rosenbach, L., Yadin, E., & Apter, A. (2010). Prolonged exposure versus dynamic therapy for adolescent PTSD: A pilot randomized controlled trial. Journal of the American Academy of Child & Adolescent Psychiatry, 49, 1034–1042.

**Goenjian, A. K.**, Karayan, I., Pynoos, R. S., Minassian, D., Najarian, L. M., Steinberg, A. M., & Fairbanks, L. A. (1997). Outcome of psychotherapy among early adolescents after trauma. American Journal of Psychiatry, 154, 536–542.

**Goldbeck, L.**, Muche, R., Sachser, C., Tutus, D., & Rosner, R. (2016). Effectiveness of Trauma-Focused Cognitive Behavioral Therapy for children and adolescents: A randomized controlled trial in eight German mental health clinics. Psychotherapy and Psychosomatics, 85(3), 159–170. <https://doi.org/10.1159/000442824>

**Gordon, J. S.**, Staples, J. K., Blyta, A., Bytyqi, M., & Wilson, A. T. (2008). Treatment of posttraumatic stress disorder in postwar Kosovar adolescents using mind-body skills groups: A randomized controlled trial. The Journal of Clinical Psychiatry, 69(9), 1469–1476. <https://doi.org/10.4088/jcp.v69n0915>

**Jaberghaderi, N.**, Greenwald, R., Rubin, A., Zand, S. O., & Dolatabadi, S. (2004). A comparison of CBT and EMDR for sexually-abused Iranian girls. Clinical Psychology & Psychotherapy, 11(5), 358–368. <https://doi.org/10.1002/cpp.395>

**Jaycox, L. H.**, Langley, A. K., Stein, B. D., Wong, M., Sharma, P., Scott, M., & Schonlau, M. (2009). Support for students exposed to trauma: A pilot study. School Mental Health, 1, 49–60.

**Jensen, T. K.**, Holt, T., Ormhaug, S. M., Egeland, K., Granly, L., Hoaas, L. C., Hukkelberg, S. S., Indregard, T., Stormyren, S. D., & Wentzel-Larsen, T. (2014). A randomized effectiveness study comparing trauma-focused cognitive behavioral therapy with therapy as usual for youth. Journal of Clinical Child & Adolescent Psychology, 43, 356–369.

**Kassam-Adams, N.**, García-España, J. F., Marsac, M. L., Kohser, K. L., Baxt, C., Nance, M., & Winston, F. (2011). A pilot randomized controlled trial assessing secondary prevention of traumatic stress integrated into pediatric trauma care. Journal of Traumatic Stress, 24(3), 252–259. <https://doi.org/10.1002/jts.20640>

**Kassam-Adams, N.**, Marsac, M. L., Kohser, K. L., Kenardy, J., March, S., & Winston, F. K. (2016). Pilot randomized controlled trial of a novel web-based intervention to prevent posttraumatic stress in children following medical events. Journal of Pediatric Psychology, 41(1), 138–148.

**Kazak, A. E.**, Alderfer, M. A., Streisand, R., Simms, S., Rourke, M. T., Barakat, L. P., … Cnaan, A. (2004). Treatment of posttraumatic stress symptoms in adolescent survivors of childhood cancer and their families: A randomized clinical trial. Journal of Family Psychology, 18(3), 493–504. <https://doi.org/10.1037/0893-3200.18.3.493>

**Kemp, M.**, Drummond, P., & McDermott, B. (2010). A wait-list controlled pilot study of eye movement desensitization and reprocessing (EMDR) for children with post-traumatic stress disorder (PTSD) symptoms from motor vehicle accidents. Clinical Child Psychology and Psychiatry, 15(1), 5–25. <https://doi.org/10.1177/1359104509339086>

**Kenardy, J.**, Thompson, K., Le Brocque, R., & Olsson, K. (2008). Information-provision intervention for children and their parents following pediatric accidental injury. European Child & Adolescent Psychiatry, 17, 316–325.

**Kenardy, J. A.**, Cox, C. M., & Brown, F. L. (2015). A web-based early intervention can prevent long-term PTS reactions in children with high initial distress following accidental injury. Journal of Traumatic Stress, 28, 366–369.

**King, N. J.**, Tonge, B. J., Mullen, P., Myerson, N., Heyne, D., Rollings, S., … Ollendick, T. H. (2000). Treating sexually abused children with posttraumatic stress symptoms: A randomized clinical trial. Journal of the American Academy of Child & Adolescent Psychiatry, 39, 1347–1355.

**Kramer, D. N.**, & Landolt, M. A. (2014). Early psychological intervention in accidentally injured children ages 2–16: A randomized controlled trial. European Journal of Psychotraumatology, 5, Article 24402. <https://doi.org/10.3402/ejpt.v5.24402>

**Langley, A. K.**, Gonzalez, A., Sugar, C. A., Solis, D., & Jaycox, L. (2015). Bounce Back: Effectiveness of an elementary school-based intervention for multicultural children exposed to traumatic events. Journal of Consulting and Clinical Psychology, 83(5), 853–865. <https://doi.org/10.1037/ccp0000051>

**Layne, C. M.**, Saltzman, W. R., Poppleton, L., Burlingame, G. M., Pasalic, A., Durakovic, E., … Pynoos, R. S. (2008). Effectiveness of a school-based group psychotherapy program for war-exposed adolescents: A randomized controlled trial. Journal of the American Academy of Child & Adolescent Psychiatry, 47, 1048–1062.

**Lieberman, A. F.**, Van Horn, P., & Ippen, C. G. (2005). Toward evidence-based treatment: Child-parent psychotherapy with preschoolers exposed to marital violence. Journal of the American Academy of Child & Adolescent Psychiatry, 44, 1241–1248.

**Lyshak-Stelzer, F.**, Singer, P., Patricia, S. J., & Chemtob, C. M. (2007). Art therapy for adolescents with posttraumatic stress disorder symptoms: A pilot study. Art Therapy, 24, 163–169.

**Marsac, M. L.**, Hildenbrand, A. K., Kohser, K. L., Winston, F. K., Li, Y., & Kassam-Adams, N. (2013). Preventing posttraumatic stress following pediatric injury: A randomized controlled trial of a web-based psycho-educational intervention for parents. Journal of Pediatric Psychology, 38(10), 1101–1111.

**McMullen, J.**, O’Callaghan, P., Shannon, C., Black, A., & Eakin, J. (2013). Group trauma-focused cognitive-behavioural therapy with former child soldiers and other war-affected boys in the DR Congo: A randomised controlled trial. Journal of Child Psychology and Psychiatry, 54(11), 1231–1241. <https://doi.org/10.1111/jcpp.12094>

**Meiser-Stedman, R.**, Smith, P., McKinnon, A., Dixon, C., Trickey, D., Ehlers, A., … Dalgleish, T. (2017). Cognitive therapy as an early treatment for post-traumatic stress disorder in children and adolescents: A randomized controlled trial addressing preliminary efficacy and mechanisms of action. Journal of Child Psychology and Psychiatry, 58, 623–633.

**Murray, L. K.**, Skavenski, S., Kane, J. C., Mayeya, J., Dorsey, S., Cohen, J. A., … Bolton, P. A. (2015). Effectiveness of Trauma-Focused Cognitive Behavioral Therapy among trauma-affected children in Lusaka, Zambia: A randomized clinical trial. JAMA Pediatrics, 169(8), 761–769. <https://doi.org/10.1001/jamapediatrics.2015.0580>

**Nugent, N. R.**, Christopher, N. C., Crow, J. P., Browne, L., Ostrowski, S., & Delahanty, D. L. (2010). The efficacy of early propranolol administration at reducing PTSD symptoms in pediatric injury patients: A pilot study. Journal of Traumatic Stress, 23(2), 282–287. https://doi.org/10.1002/jts.20517

**O’Callaghan, P.**, McMullen, J., Shannon, C., Rafferty, H., & Black, A. (2013). A randomized controlled trial of trauma-focused cognitive behavioral therapy for sexually exploited, war-affected Congolese girls. Journal of the American Academy of Child & Adolescent Psychiatry, 52(4), 359–369. <https://doi.org/10.1016/j.jaac.2013.01.013>

**O’Callaghan, P.**, McMullen, J., Shannon, C., & Rafferty, H. (2015). Comparing a trauma focused and non trauma focused intervention with war affected Congolese youth. Intervention, 13(1), 28–44. https://doi.org/10.1097/WTF.0000000000000054

**Pfeiffer, E.**, Sachser, C., Rohlmann, F., & Goldbeck, L. (2018). Effectiveness of a trauma-focused group intervention for young refugees: A randomized controlled trial. Journal of Child Psychology and Psychiatry, 59, 1171–1179.

**Pityaratstian, N.**, Piyasil, V., Ketumarn, P., Sitdhiraksa, N., Ularntinon, S., & Pariwatcharakul, P. (2015). Randomized controlled trial of group cognitive behavioural therapy for post-traumatic stress disorder in children and adolescents exposed to tsunami in Thailand. Behavioural and Cognitive Psychotherapy, 43(5), 549–561. <https://doi.org/10.1017/S1352465813001197>

**Prchal, A.**, Graf, A., Bergstraesser, E., & Landolt, M. A. (2012). A two-session psychological intervention for siblings of pediatric cancer patients: A randomized controlled pilot trial. Child and Adolescent Psychiatry and Mental Health, 6(1), Article 3. <https://doi.org/10.1186/1753-2000-6-3>

**Robb, A.**, Cueva, J., Sporn, J., Yang, R., & Vanderburg, D. (2010). Sertraline treatment of children and adolescents with posttraumatic stress disorder: A double-blind, placebo-controlled trial. Journal of Child and Adolescent Psychopharmacology, 20, 463–471.

**Rossouw, J.**, Yadin, E., Alexander, D., Mbanga, I., Jacobs, T., & Seedat, S. (2016). A pilot and feasibility randomised controlled study of Prolonged Exposure Treatment and supportive counselling for post-traumatic stress disorder in adolescents: A third world, task-shifting, community-based sample. Trials, 17, Article 548. <https://doi.org/10.1186/s13063-016-1677-6>

**Rossouw, J.**, Yadin, E., Alexander, D., & Seedat, S. (2018). Prolonged exposure therapy and supportive counselling for post-traumatic stress disorder in adolescents: Task-shifting randomised controlled trial. British Journal of Psychiatry, 213, 587–594.

**Ruf, M.**, Schauer, M., Neuner, F., Catani, C., Schauer, E., & Elbert, T. (2010). Narrative exposure therapy for 7- to 16-year-olds: A randomized controlled trial with traumatized refugee children. Journal of Traumatic Stress, 23(4), 437–445. <https://doi.org/10.1002/jts.20548>

**Runyon, M. K.**, Deblinger, E., & Steer, R. A. (2010). Group cognitive behavioral treatment for parents and children at-risk for physical abuse: An initial study. Child & Family Behavior Therapy, 32(3), 196–218. <https://doi.org/10.1080/07317107.2010.500515>

**Salloum, A.**, Wang, W., Robst, J., Murphy, T. K., Scheeringa, M. S., Cohen, J. A., & Storch, E. A. (2016). Stepped care versus standard trauma-focused cognitive behavioral therapy for young children. Journal of Child Psychology and Psychiatry, 57(5), 614–622. <https://doi.org/10.1111/jcpp.12471>

**Scheeringa, M. S.**, Weems, C. F., Cohen, J. A., Amaya-Jackson, L., & Guthrie, D. (2011). Trauma-focused cognitive-behavioral therapy for posttraumatic stress disorder in 3–6 year-old children: A randomized clinical trial. Journal of Child Psychology and Psychiatry, 52, 853–860.

**Schottelkorb, A. A.**, Doumas, D. M., & Garcia, R. (2012). Treatment for childhood refugee trauma: A randomized, controlled trial. International Journal of Play Therapy, 21(2), 57–73. <https://doi.org/10.1037/a0027430>

**Shein-Szydlo, J.**, Sukhodolsky, D. G., Kon, D. S., Tejeda, M. M., Ramirez, E., & Ruchkin, V. (2016). A randomized controlled study of cognitive-behavioral therapy for posttraumatic stress in street children in Mexico City. Journal of Traumatic Stress, 29(5), 406–414. <https://doi.org/10.1002/jts.22124>

**Smith, P.**, Yule, W., Perrin, S., Tranah, T., Dalgleish, T., & Clark, D. M. (2007). Cognitive-behavioral therapy for PTSD in children and adolescents: A preliminary randomized controlled trial. Journal of the American Academy of Child & Adolescent Psychiatry, 46, 1051–1061.

**Stallard, P.**, Velleman, R., Salter, E., Howse, I., Yule, W., & Taylor, G. (2006). A randomised controlled trial to determine the effectiveness of an early psychological intervention with children involved in road traffic accidents. Journal of Child Psychology and Psychiatry, 47, 127–134.

**Stein, B. D.**, Jaycox, L. H., Kataoka, S. H., Wong, M., Tu, W., Elliott, M. N., & Fink, A. (2003). A mental health intervention for schoolchildren exposed to violence: A randomized controlled trial. JAMA, 290(5), 603–611. <https://doi.org/10.1001/jama.290.5.603>

**Zehnder, D.**, Meuli, M., & Landolt, M. (2010). Effectiveness of a single-session early psychological intervention for children after road traffic accidents: A randomised controlled trial. Child and Adolescent Psychiatry and Mental Health, 4(7). https://capmh.biomedcentral.com/articles/10.1186/1753-2000-4-7
